# Diffusion tensor imaging of dolphin brains reveals direct auditory pathway to temporal lobe

**DOI:** 10.1098/rspb.2015.1203

**Published:** 2015-07-22

**Authors:** Gregory S. Berns, Peter F. Cook, Sean Foxley, Saad Jbabdi, Karla L. Miller, Lori Marino

**Affiliations:** 1 Psychology Department, Emory University, Atlanta, GA, USA; 2 FMRIB Centre, University of Oxford, Oxford, UK; 3 The Kimmela Center for Animal Advocacy, Kanab, UT, USA

**Keywords:** diffusion tensor imaging, dolphin, auditory

## Abstract

The brains of odontocetes (toothed whales) look grossly different from their terrestrial relatives. Because of their adaptation to the aquatic environment and their reliance on echolocation, the odontocetes' auditory system is both unique and crucial to their survival. Yet, scant data exist about the functional organization of the cetacean auditory system. A predominant hypothesis is that the primary auditory cortex lies in the suprasylvian gyrus along the vertex of the hemispheres, with this position induced by expansion of ‘associative′ regions in lateral and caudal directions. However, the precise location of the auditory cortex and its connections are still unknown. Here, we used a novel diffusion tensor imaging (DTI) sequence in archival post-mortem brains of a common dolphin (*Delphinus delphis*) and a pantropical dolphin (*Stenella attenuata*) to map their sensory and motor systems. Using thalamic parcellation based on traditionally defined regions for the primary visual (V1) and auditory cortex (A1), we found distinct regions of the thalamus connected to V1 and A1. But in addition to suprasylvian-A1, we report here, for the first time, the auditory cortex also exists in the temporal lobe, in a region near cetacean-A2 and possibly analogous to the primary auditory cortex in related terrestrial mammals (Artiodactyla). Using probabilistic tract tracing, we found a direct pathway from the inferior colliculus to the medial geniculate nucleus to the temporal lobe near the sylvian fissure. Our results demonstrate the feasibility of post-mortem DTI in archival specimens to answer basic questions in comparative neurobiology in a way that has not previously been possible and shows a link between the cetacean auditory system and those of terrestrial mammals. Given that fresh cetacean specimens are relatively rare, the ability to measure connectivity in archival specimens opens up a plethora of possibilities for investigating neuroanatomy in cetaceans and other species.

## 1. Introduction

Cetaceans—especially toothed whales, dolphins and porpoises—have intrigued the public and scientific communities for decades. Cetaceans are cognitively complex and have rich social lives, but little is known about how their brains function, especially when compared with their terrestrial relatives. The cetacean brain has been relatively inaccessible because of its size, difficulties in obtaining fresh specimens and the welfare considerations that rule out invasive recordings or tracer studies. But the advent of neuroimaging, especially MRI, has provided a new set of possibilities for understanding cetacean brain organization in non-invasive ways.

Several MRI investigations have demonstrated the complex anatomy of cetacean brains, providing detailed *in situ* images amenable to quantitative analyses of the size and morphology of brain structures [1–6]. To further understand the relationship of structure to function, however, information is needed about how different brain regions are connected to each other and how this information relates to specific cognitive or perceptual processes [7]. Historically, the gold standard for mapping connectivity was a tracer study. The technique required the injection of either an anterograde or retrograde tracer into specific brain regions, followed by the euthanization of the animal for sectioning of the brain. Given that most people now find this approach ethically objectionable in whales and dolphins and strict legal protections for marine mammals limit the use of invasive techniques, new non-invasive methods are needed to measure brain connectivity in cetaceans. Diffusion tensor imaging (DTI) has been used with great success in humans and other primates to map white-matter pathways [8] and is qualitatively correlated with tracts identified with tracers [9]. Although DTI has been widely deployed as a tool for comparative neurobiology, especially in primates [10,11], it has not yet been used in marine mammals.

There are significant challenges to using DTI in the brain of a cetacean. Because cetaceans are fully aquatic, fresh specimens are rare, and those that do exist are usually obtained opportunistically from stranding events. As such, existing specimens may be old and have undergone degradation. Tissue relaxation properties (T1 and T2) change over time [12], and conventional DTI sequences, which are designed for living tissue (or freshly preserved), are not optimized for these types of specimens. However, novel DTI sequences based on steady-state free precession (SSFP) have recently been shown to be more efficient for imaging of post-mortem human brains [13]. Here, we demonstrate its efficacy in delineating the auditory pathways of the dolphin brain from archival specimens preserved over a long period of time.

Dolphins, which belong to the suborder of toothed whales (odontocetes), use high-frequency click trains, whistles and burst-pulses for a variety of purposes, such as echolocation, communication, navigation and foraging [14–16]. Not surprisingly, they have complex auditory systems, but there is much debate about the location of the auditory cortex. Based on early electrophysiological studies, a predominant hypothesis is that the primary auditory cortex is located in the suprasylvian gyrus along the vertex of the hemispheres [17,18], lateral and adjacent to the primary visual cortex. A single set of tracer studies seemed to confirm this and hinted at the possibility of temporal lobe projections but was not definitive because no injections were performed in the deep temporal lobe [19]. The dorsal location of the auditory cortex is strikingly different from terrestrial mammals, including the closest taxa, artiodactyls (even-toed ungulates) [20]. It has been hypothesized that in the course of odontocete evolution the auditory cortical regions were displaced to their current unusual position by expansion of ‘associative’ regions in the lateral (temporal) and caudal directions [21–23]. However, the definitive location of the auditory cortex is still debated, and the mapping of sensory regions in the dolphin suprasylvian cortex has met with some speculation about whether these regions are the only auditory cortical areas in the dolphin brain. Ridgway noted that the dorsal cortex of dolphins is more accessible to electrophysiological study than deeper regions, and he conjectured that electrode placement in these earlier studies may have been too shallow to detect additional auditory processing regions in the temporal cortex of dolphins [24], thus preventing identification of deeper auditory cortical regions, but this proposition has had to wait for appropriate methods to measure connectivity in the dolphin brain.

## 2. Material and methods

### (a) Specimens

The primary specimen was the post-mortem brain of an adult female, pregnant, common dolphin (*Delphinus delphis*) which stranded dead in February 2001 at Buxton, North Carolina (Field #PTM135), with approximately seven other live common dolphins which eventually returned to the sea. The carcass was in fresh condition (Smithsonian Condition Code 2) with no evidence of damage [25]. Total body length was 203 cm and total body weight was 83 kg. The brain was extracted from the skull approximately 24 h after the dolphin was discovered. It was weighed and placed in 10% neutral buffered formalin 62 days prior to an initial MRI study of gross neuroanatomy [3]. Fresh brain weight was 981 g. The specimen measured 132 mm in anterior–posterior length, 155 mm in bitemporal width and 96 mm in height.

For confirmation of tractography, a second specimen was also scanned. This was the post-mortem brain of an adult female spotted dolphin (*Stenella attenuata*) which stranded freshly post-mortem at Camp Lejeune in North Carolina (WAM576). The total body length of the specimen was 191 cm, and the total body mass was 57 kg. The specimen was necropsied and the brain was collected while still fresh. Both specimens were kept in fluid (10% neutral buffered formalin changed on a regular basis) at Emory University until its current use.

For the current imaging session, the specimens were set in 2% agarose (Phenix Research Products Low EEO Molecular Biology Grade Agarose) doped with an insoluble mixture of 2 mM gadolinium (III) oxide (Acros Organics, Fisher Scientific) [26].

### (b) Imaging

All imaging was performed on a 3 T Siemens Trio with standard gradients and a 32-channel head receive coil. DTI was acquired with a diffusion-weighted (DW) SSFP sequence [13]. Although DW-SSFP is more motion-sensitive than the usual DW spin-echo sequences used for *in vivo* DTI, DW-SSFP is more SNR efficient for tissues with short T2, which is critical for post-mortem imaging. We acquired one set of DW-SSFP images weighted along 52 directions (FOV = 166 mm, voxel size = 1.3 mm isotropic, TR = 31 ms, TE = 24 ms, flip angle = 29°, bandwidth = 159 Hz pixel^−1^, *q* = 255 cm^−1^, *G*_max_ = 38 mT m^−1^, gradient duration = 15.76 ms). Six images were acquired with these same parameters except with *q* = 10 cm^−1^ applied in one direction only (these serve as a signal reference similar to *b* = 0 scans in conventional spin-echo acquisitions). Proper modelling of the DW-SSFP signal requires knowledge of T1 and T2 values, which are drastically altered in post-mortem compared with *in vivo* tissue. These values were calculated based on a series of T1-weighted images (TIR sequence with TR = 1000 ms, TE = 12 ms and TI = 30, 120 and 900 ms) and T2-weighted images (TSE sequence with TR = 1000 ms and TE = 14, 29 and 43 ms). Structural images were acquired using a three-dimensional balanced SSFP sequence (TR = 7.03 ms, TE = 3.52 ms and flip angle = 37°). Balanced SSFP images were acquired in pairs with the RF phase incrementing 0° and 180°, which were averaged later to reduce banding artefacts [13,27]. This yielded structural images with 0.6 × 0.6 × 0.5 mm resolution. It took approximately 8 h to acquire all scans.

All images were processed with FSL tools modified to account for the DW-SSFP signal model and which incorporated tissue T1 and T2, which were estimated to be 350 and 50 ms, respectively, for white matter in these specimens. This yielded a *b*_eff_ ≈ 3500 s mm^−2^ for the diffusion-weighted scans [28]. All diffusion images were registered to one *q* = 10 cm^−1^ reference image, and the references were averaged together to create a mean reference. To fit a diffusion tensor model at each voxel, we fitted an extension of the model proposed by Buxton [29] to incorporate Gaussian (DTI) anisotropic diffusion [30]. The tensor parameters (three eigenvalues, three orientations) were estimated using the Metropolis Hastings algorithm with a positivity constraint of the tensor eigenvalues. This yielded estimates for fractional anisotropy, mean diffusivity and three eigenvectors representing the principal directions of the diffusion tensor [30]. For tractography, we used a similarly modified version of BEDPOSTX that incorporated the new signal model [27–29] with two crossing fibres per voxel but otherwise default options [31]. These calculations took approximately 2 days on a dual Xeon processor with 16 cores running in parallel.

For visualization, we used deterministic tractography as implemented in DSI Studio [32]. This algorithm estimates the orientation distribution functions (ODFs) by first filtering out noisy fibres based on a quantitative anisotropy (QA) metric. This approach removes crossing fibres that might otherwise derail tract tracing and seems relatively insensitive to partial volume effects. We used this approach primarily for visualization and as a complement to the probabilistic methods implemented in FSL. For whole-brain rendering, we used the following parameters: QA threshold = 0.09, angular threshold = 55°, step size = 0.55 mm and minimum length = 10 mm. Three-dimensional images of tracks were rendered at 1° increments of rotation of the brain and assembled into a movie. The ODF estimated in DSI Studio is theoretically only a valid fibre ODF in the case of standard pulsed-gradient spin-echo diffusion experiments, and may not represent the true ODF for SSFP diffusion experiments. However, for the purposes of tractography, we only use the ODF peaks, not the full ODF shape, and therefore, this model-free ODF approach remains a valid approach even for SSFP diffusion.

### (c) Tractography

Because of the importance of the dolphin's auditory system for both communication and echolocation, we focused our attention on auditory pathways. However, the auditory cortex is potentially very large in the dolphin. To guide subsequent analyses, we began by placing two bilateral virtual ‘seeds' in the inferior colliculi (IC). The IC are large and well defined in the dolphin brain, and because all ascending auditory information passes through them, they are easily identified anatomically, thus serving as an ideal starting point to delineate the auditory pathways to the cerebral cortex. We drew a region of interest (ROI) encompassing each whole IC to act as seeds for probabilistic tract tracing [8,31]. For every voxel in this seed mask, a series of ‘streamlines' was calculated that formed a representation of the likely tract structure by incorporating the underlying uncertainty in the preferred diffusion of water, which occurs along the predominate orientation of fibres passing through a voxel [13]. By proceeding from voxel to voxel, one can trace specific fibre tracts. We used the default 5000 streamlines from each voxel, a step length of 0.5 mm, distance correction and a curvature threshold of 88°. For parameter sensitivity analysis, we repeated the tractography with angular thresholds of 80° and 45°.

Probabilistic tractography identifies the most likely pathways from a seed location but does not necessarily exclude the possibility of other tracts. Previous electrophysiological work, however, had located the putative primary auditory cortex in a broad swath of the suprasylvian gyrus (suprasylvian-A1) extending from the posterior portion of the brain to the vertex [2,17,19,23]. To determine whether a pathway existed between the thalamus and suprasylvian-A1, we performed thalamic parcellation [33] based on three cortical ROIs: (i) visual cortex (V1) located medial to the lateral gyrus; (ii) suprasylvian-A1; and (iii) deep temporal lobe. Suprasylvian-A1 and V1 were drawn in the coronal and transverse planes and were modelled after the canonical definitions of these regions [23]. Suprasylvian-A1 was drawn on the suprasylvian gyrus in the cortex just lateral to the lateral sulcus, and extending partway laterally to the suprasylvian sulcus. The rostral boundary was set at the approximate vertex of the cortex, in a plane coronal with the medial-caudal boundary of the left and right thalamus. The inferior caudal boundary was set near the caudal-most curve of the cortex, in a plane transversely with the rostral-inferior boundary of the anterior corpus callosum. V1 was drawn on the lateral gyrus, just medial to the lateral sulcus. The rostral boundary was set caudal to the cortical apex, in a plane transverse with the superior rostral boundary of the lateral anterior corpus callosum. The inferior caudal boundary was set at the same level as for suprasylvian-A1. The deep temporal regions surrounding the IC projections bilaterally through medial geniculate nucleus (MGN) were drawn in the coronal plane. The superior boundaries were just dorsal to the superior boundaries of the ICs. The inferior boundaries were just ventral to the inferior boundaries of the ICs. Medial boundaries were set at the level of the dominant inferior projections from MGN via IC, and lateral boundaries were arbitrarily defined approximately 1 cm lateral to this.

Exploratory analyses were performed on the orbital (‘frontal’) lobes and their connections to the basal ganglia. Five bilateral orbital regions were defined arbitrarily in the coronal plane by tracing the cortex around the five predominant white-matter protrusions. The caudal boundary for these masks was the postcruciate sulcus, and they proceeded to the rostral extent of the brain.

## 3. Results

**Figure 1.**
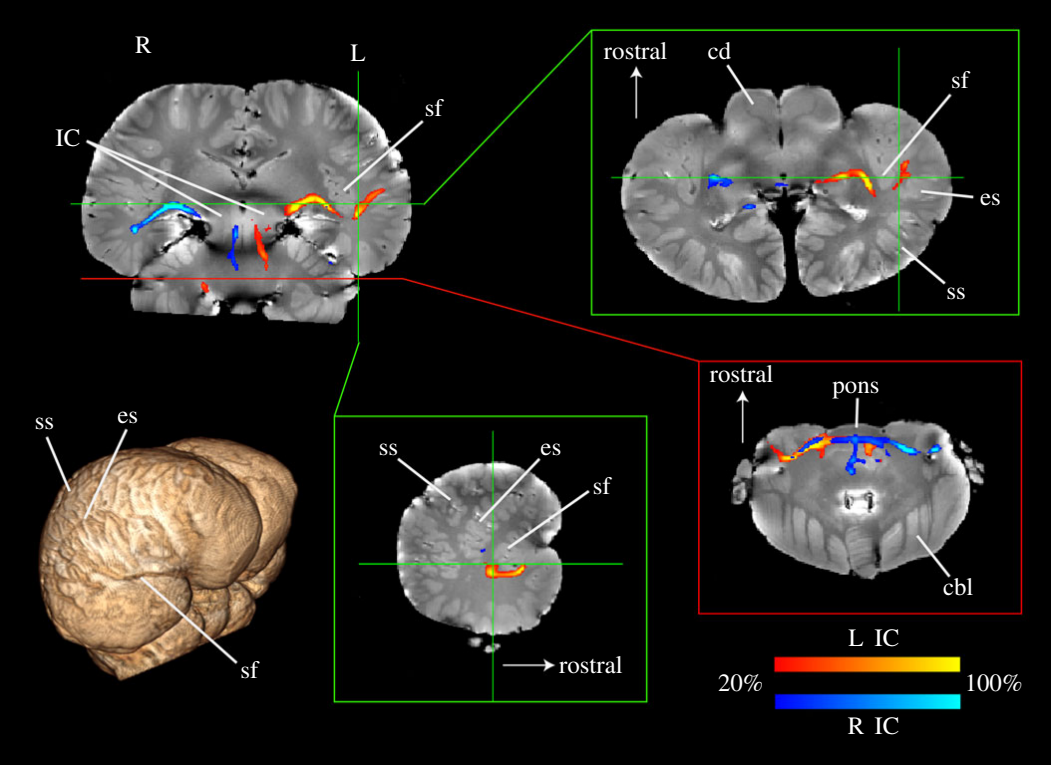
Probabilistic tractography from IC in a common dolphin brain (*Delphinus delphis*). Using seeds drawn on the left (red) and right (blue) inferior colliculi (IC, upper left), a tract to the temporal lobe was identified, which passed through the ventrocaudal thalamus and the medial geniculate nucleus (MGN). The tract colour indicates the percentage of streamlines passing through a voxel and is thresholded such that voxels in which less than 20% of the streamlines pass are not shown. Insets show transverse slices at the level of the MGN (upper right) and cochlear nuclei (lower right) which shows the decussation of the auditory tract and entrance of the auditory nerves, and a sagittal slice through the left sylvian fissure (lower middle). Three-dimensional rendering (lower left) shows major landmarks. IC, inferior colliculus; sf, sylvian fissure; ss, suprasylvian sulcus; es, ectosylvian sulcus; cd, caudate; cbl, cerebellum.

With the IC seeds, we found a pathway to the ventrocaudal portion of the thalamus, which then turned laterally and terminated deep in the temporal lobe, near the sylvian fissure (figure 1). In the other direction, the fibres proceeded through the brainstem to the lateral lemniscus, superior olive and bilateral cochlear nuclei, confirming that we had identified the primary/ascending auditory pathway [23]. The tissue parameters of the second specimen (*Stenella attenuata*) indicated a shorter T1 of white matter (approx. 280 ms) than the *Delphinus*, suggesting more degradation had occurred in the *Stenella* with concomitant effects on the SNR of the diffusion images and a lower *b*_eff_. Nevertheless, we confirmed the existence of these same pathways in the *Stenella* by seeding the IC and visualizing pathways to the MGN and temporal lobes, albeit at a lower threshold (electronic supplementary material, figure S1).

**Figure 2.**
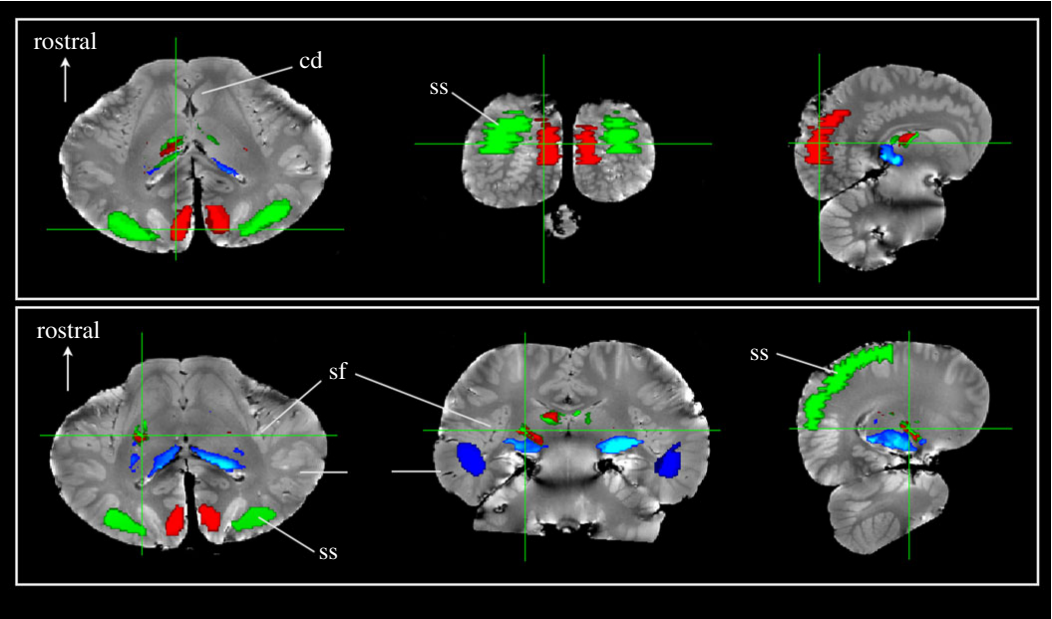
Thalamic parcellation of *Delphinus delphis* based on cortical regions. Three regions of interest were defined: (i) visual cortex (red); (ii) auditory cortex based on traditional boundaries in the suprasylvian gyrus (green) and (iii) auditory region temporal cortex based on IC tractography (blue). Seeds within the entire thalamus were traced to these regions and thresholded above 20 000 streamlines. Each row shows a set of slices that correspond to a single cursor location. The upper row is located posteriorly through the cortex while the lower row is located through thalamus. The thalamic parcellations to the three regions demonstrate that the temporal region is primarily connected to the ventrocaudal region, presumably near the medial geniculate nucleus. The visual and auditory regions overlap substantially in the thalamus and are located more dorsally and rostrally.

**Figure 3.**
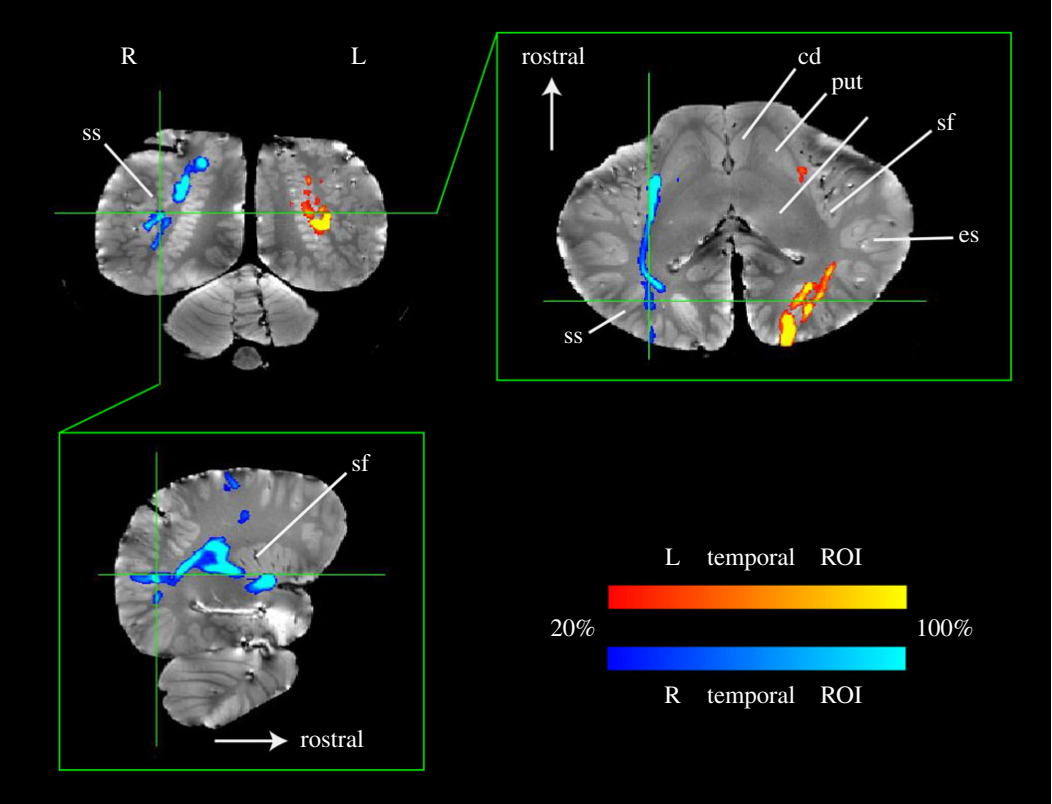
Probabilistic tractography from temporal ROIs in *Delphinus delphis*. Using seeds drawn on the left (red) and right (blue) temporal ROIs, tracts to the suprasylvian-A1 were identified. A ‘waypoint’ mask for suprasylvian-A1 was used to constrain the search space for tracts emanating from the temporal ROIs. The tract colour indicates the percentage of streamlines passing through a voxel and is thresholded such that voxels in which less than 20% of the streamlines pass are not shown.

Thalamic parcellation confirmed earlier electrophysiological findings that portions of the thalamus were, indeed, connected to suprasylvian-A1 (figure 2, green). The suprasyvian-A1 thalamic connections were located dorsal and rostral to the regions connected to the temporal lobe, which were located ventrocaudally, near the presumed location of the MGN (figure 2, blue). The V1 thalamic connections (red) had extensive overlap with the suprasylvian-A1 connections. However, our new analysis also indicated pathways between the deep temporal lobe and suprasylvian-A1 regions bilaterally (figure 3).

**Figure 4.**
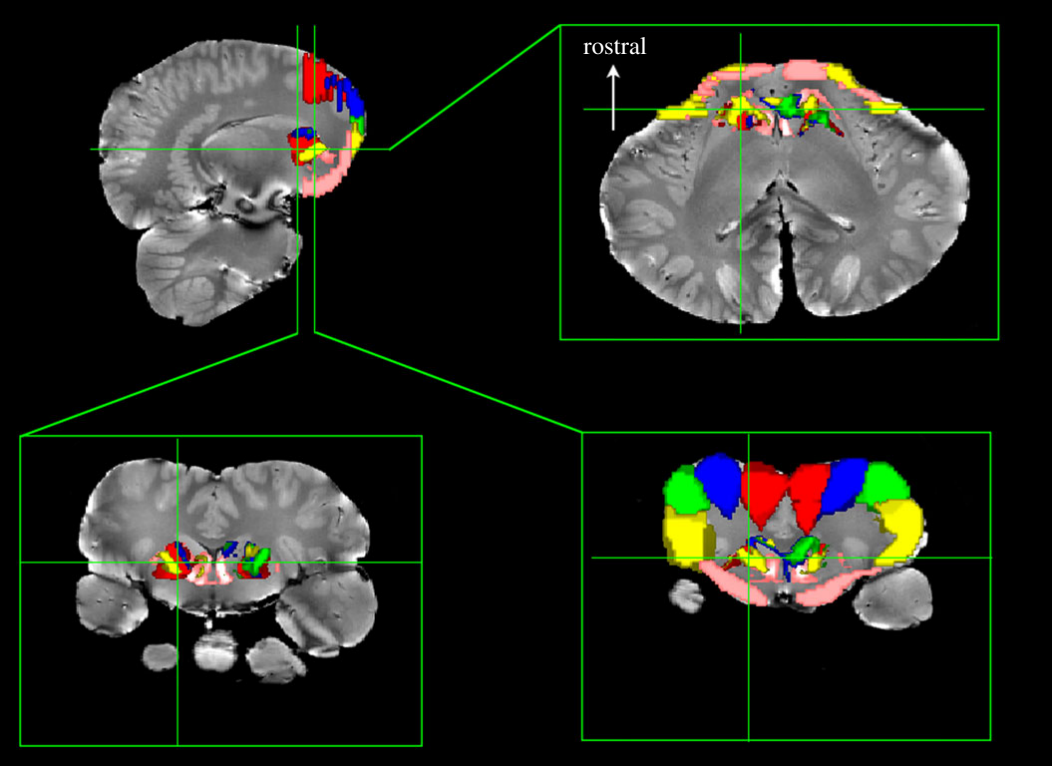
Basal ganglia parcellation based on cortical regions in *Delphinus delphis*. Five regions were defined in the orbital (‘frontal’) lobes from the longitudinal fissure (red) progressing laterally and ventrally (blue, green and yellow) to the ventral orbital lobe (pink). Seeds within the entire basal ganglia were traced to these regions and thresholded above 20 000 streamlines. Bottom row shows two coronal slices through different parts of the basal ganglia. The dorsal portions of the basal ganglia are connected to the dorsal portions of the cortex (red, blue and green), while the ventral basal ganglia are connected to the ventrolateral (yellow) and ventral orbital lobe (pink).

To confirm that the thalamic parcellation and temporal auditory pathway were not a spurious result of DTI or probabilistic tractography, we also performed a similar parcellation of the basal ganglia based on connections to the orbital lobes (figure 4). This technique has been used in humans, and a rostrocaudal gradient has been observed with the ventral portions of the striatum connected to the medial and orbital cortices, while the dorsal striatum is connected to the premotor and motor cortices [34]. We found the same rostrocaudal gradient in the dolphin brain. Notably, the most ventral parts of the orbital lobe were connected to the most ventral portions of the dolphin striatum, while the most dorsal portions of the orbital lobe—presumably the motor and premotor cortices—were connected to the dorsal striatum. This provides further evidence for the validity of the tractography methods used in this study and demonstrates that the cetacean orbital lobes are connected to the basal ganglia in a pattern similar to the connectivity of frontal lobes to basal ganglia in primates.

Whole-brain rendering showed that the hemispheres were dominated by intra-hemispheric fibres both rostrocaudally and dorsoventrally (electronic supplementary material, video S1). Consistent with previous structural MRIs of cetacean brains [2,3], we found a thin corpus callosum with apparently few fibres relative to the volume of the hemispheres. Fibres passing through the internal capsule were prominent and could be visualized projecting to/from the orbital lobes. These fibre tracts served as useful landmarks for defining the orbital lobes and motor cortex. In the brainstem, we noted a massive bundle of crossing fibres in the pons, and the optic chiasm could be seen just ventral to that. Caudal to the pons, a number of cranial nerves emerged laterally, including the large auditory nerve.

## 4. Discussion

This non-invasive study is the first to offer evidence for a direct auditory pathway from the thalamus to the temporal lobe in the cetacean brain, a pathway that has been hypothesized, but not definitively demonstrated until now. This finding has important implications for our understanding of cetacean brain evolution. It provides empirical evidence which allows for important refinements to existing models of the cetacean auditory cortex. One theory is that through the course of evolution, the cetacean auditory cortex (suprasylvian-A1) was displaced dorsally to the vertex of the brain as the temporal lobe expanded in the opposite direction [21–23]. This type of expansion would stand in contrast to the bulk of data in terrestrial mammals, showing the primary auditory cortex ventral to the suprasylvian sulcus [35], but the theory of cetacean cortical expansion has been difficult to verify without more detailed comparative data from both extant and extinct related species. The use of post-mortem connectivity data adds a new tool to the study of how brain regions expanded and were displaced through evolution. Our findings add to the theory of cetacean cortical expansion by showing that there is an additional ascending auditory tract to the temporal lobe near the previously reported location of cetacean-A2 [18].

There is still much to clarify about these findings and how they compare with brain auditory systems in other taxonomic groups and, ultimately, how they contribute to a fuller understanding of central auditory processing in cetaceans. There are some interesting comparative data from a study of temporoparietal cytoarchitecture in several cetacean species revealing that the patterns of cytoarchitectonic fields just above and below the sylvian cleft are reminiscent of the organization of the visual and auditory primary fields in carnivores, like cats [36]. However, as noted, the cetacean primary auditory cortex has been functionally localized to the suprasylvian gyrus only and it is therefore uncertain how the cytoarchitecture in the temporal lobe relates to auditory regions in other mammals.

Further depth and clarity may be brought to the present findings by exploring the brains of the closely related taxonomic group, Ungulata, and, in particular, artiodactyls (even-toed ungulates). The neocortex of cetaceans shares several cyto- and chemoarchitectural features with that of large terrestrial artiodactyls [37] consistent with the phylogenetic closeness of these species [20]. Recent morphological and molecular evidence suggests that modern Hippopotamidae are the closest extant artiodactyl relatives of cetaceans [38,39]. The only histological and morphometric study of the brain of the hippopotamus (*Hexaprotodon liberiensis*) characterized the cytoarchitecture of the temporal lobe in this species and revealed both similarities and differences in cellular morphology and distribution with cetacean brains [40]. Unfortunately, it did not provide any tractography information on connectivity patterns which could be compared with the present findings. In a very early electrophysiological study of several ungulate species (goats, sheep, pigs and horses) the primary auditory region was identified in a region of the temporal lobe shared with many other mammalian species, namely ventral to the suprasylvian sulcus [41]. A more recent electrophysiology study in pigs confirmed the location of the auditory cortex to be centred around the sylvian fissure and ventral to the suprasylvian sulcus [42]. A volumetric study of both wild boars and domestic pigs also identified the auditory cortex ventral to the suprasylvian sulcus [43]. Thus, in many ungulates, of which the pig is the most studied, the auditory cortex is located ventral to the suprasylvian sulcus.

Although the artiodactyls are the closest extant species, the brains of cetaceans are still markedly different in gross morphology from ungulates. Indeed, the boundaries of what may be considered the ‘temporal lobe’ may be so different that the term ceases to be useful for comparative purposes. Salient landmarks for the auditory cortex in artiodactyls include the sylvian fissure and the suprasylvian sulcus, and the newly identified dolphin auditory region lies at the caudal end of the sylvian fissure ventral to the suprasylvian sulcus, not too distant from the previously termed cetacean-A2 [18]. However, it must be emphasized that very little data exist to confirm the character of ‘A2’ in cetaceans. Moreover, the relative sizes, i.e. expansion, of A1 and A2 do not give a direct interpretation of function, which depends more generally on how regions are connected to each other [44,45]. Patterns of connectivity may be a more parsimonious framework of understanding brain evolution and function than the sizes of brain regions, i.e. the principle of proper mass [46].

Although DTI is not a perfect measure of white-matter connectivity [47], it does well at capturing major fibre tracts to a degree that meaningful comparisons across species can be performed [9–11]. There are, of course, limitations. As shown here, tractography is primarily qualitative. The size of the tracts depends on a number of parameters, including the size of the seed region, the number of streamlines and angular threshold. We investigated sensitivity to all of these parameters and, not surprisingly, found that as each was decreased, the number of streamlines decreased. Regardless of threshold, however, the dominant ascending pathways from the IC passed through the caudal thalamus and laterally toward the temporal lobes. The second specimen (*Stenella attenuata*) showed the same pathways, albeit at a lower a threshold, suggesting that this specimen was not preserved as well as the *Delphinus*. Considering each inferior colliculus as an independent seed, all four colliculi were found to exhibit paths to the ipsilateral deep temporal lobe. This strongly suggests that these results are not artefactual.

Together, these findings are consistent with an interpretation of suprasylvian-A1 as a separate auditory processing region. The overlap between connections to V1 and A1 suggest, tentatively, that this may be an auditory region functionally connected to vision in the context of echolocation. This arrangement would be strikingly similar to the cortical organization of terrestrial echolocators, namely bats, in which thalamocortical projections feed at least three discrete ranging areas [48], and in which genome-wide associations in terrestrial and aquatic echolocators have suggested convergent evolution of genes linked to hearing and vision [49]. Because DTI does not yield directional information of the pathways, we cannot determine whether the pathway to suprasylvian-A1 splits off from the primary thalamocortical pathway or whether it represents a cortico-cortical pathway from temporal lobe, or even if they represent corticothalamic back projections as in the bat. If suprasylvian-A1 is, indeed, involved in echolocation, there should be differences in this region between echolocators (odontocetes) and non-echolocating whales (mysticetes). Indeed, the brain of one mysticete, a humpback whale, has been investigated and demonstrated both similarities and differences at the cytoarchitectural level [36,50]. Future DTI studies that concentrate on this region, potentially at higher resolution, may resolve this question.

The present findings are the first to demonstrate that a putative auditory afferent system from the thalamus to the temporal lobe exists in cetaceans. Although, further interpretation of the present findings await more data about both cetacean and Artiodactyla brains, these results compellingly point to the possibility of a future revision of our knowledge of how cetaceans process auditory and, perhaps, other forms of sensory information. More generally, we have demonstrated that DTI can be used to investigate connectivity in post-mortem cetacean brains—even in archival specimens more than a decade old. This is important because fresh specimens are rare. The ability to measure connectivity in archival specimens opens up a plethora of possibilities for investigating neuroanatomy in cetaceans and other rare species. Although there have been several studies of cetacean neuroanatomy based on MRI, the ability to examine connectivity in cetacean brains has, until now, been elusive. Here, we have provided proof of concept that DTI has the potential to give further insights into the relatively inaccessible cetacean brain and allow better-informed inferences about the cognitive adaptations that have shaped one of the most intriguing taxa in the mammalian world.

## Supplementary Material

Fig. S1. Probabilistic tractography from inferior colliculi of a pantropical dolphin (Stenella attenuata).

## Data accessibility

The following data are available for each specimen in NIFTI format in the Dryad repository (https://datadryad.org/resource/doi:10.5061/dryad.51s8h): structural image, average B0 image and bedpostX files for tractography.

## References

[1] Oelschläger HHA Haas-Rioth M Fung C Ridgway SH Knauth M 2008 Morphology and evolutionary biology of the dolphin (*Delphinus* sp.) brain—MR imaging and conventional histology. Brain Behav. Evol. 71, 68–86. (10.1159/000110495) 17975302

[2] Oelschlager HHA Ridgway SH Knauth M 2010 Cetacean brain evolution: dwarf sperm whale (*Kogia sima*) and common dolphin (*Delphinus delphis*)—an investigation with high-resolution 3D MRI. Brain Behav. Evol. 75, 33–62. (10.1159/000293601) 20203478

[3] Marino L Sudheimer KD Pabst DA McLellan WA Filsoof D Johnson JI 2002 Neuroanatomy of the common dolphin (*Delphinus delphis*) as revealed by magnetic resonance imaging. Anat. Rec. 268, 411–429. (10.1002/ar.10181) 12420290

[4] Montie EW Ketten DR Schneider G Marino L Touhey KE Hahn ME 2007 Neuroanatomy of the subadult and fetal brain of the Atlantic white-sided dolphin (*Lagenorhynchus acutus*) from *in situ* magnetic resonance images. Anat. Rec. 290, 1459–1479. (10.1002/ar.20612) 17957751

[5] Marino L Murphy TL DeWeerd AL Morris JA Ridgway SH Fobbs AJ Humblot N Johnson JI 2001 Anatomy and three-dimensional reconstructions of the brain of a white whale (*Delphinapterus leucas*) from magnetic resonance images (MRI). Anat. Rec. 262, 429–439. (10.1002/ar.1051) 11275973

[6] Marino L Sherwood CC Tang CY Delman BN Naidich TP Johnson JI Hof PR 2004 Neuroanatomy of the killer whale (*Orcinus orca*) from magnetic resonance imaging. Anat. Rec. 281A, 1256–1263. (10.1002/ar.a.20075) 15486954

[7] Krubitzer L Kaas J 2005 The evolution of the neocortex in mammals: how is phenotypic diversity generated? Curr. Opin. Neurobiol. 15, 444–453. (10.1016/j.conb.2005.07.003) 16026978

[8] Behrens TEJ Woolrich MW Jenkinson M Johansen-Berg H Nunes RG Clare S Matthews PM Brady JM Smith SM 2003 Characterization and propagation of uncertainty in diffusion-weighted MR imaging. Magn. Reson. Med. 50, 1077–1088. (10.1002/mrm.10609) 14587019

[9] Jbabdi S Lehman JF Haber SN Behrens TE 2013 Human and monkey ventral prefrontal fibers use the same organizational principles to reach their targets: tracing versus tractography. J. Neurosci. 33, 3190–3201. (10.1523/JNEUROSCI.2457-12.2013) 23407972 PMC3602794

[10] Mars RB Neubert F-X Verhagen L Sallet J Miller KL Dunbar RIM Barton RA 2014 Primate comparative neuroscience using magnetic resonance imaging: promises and challenges. Front. Neurosci. 8, 298 (10.3389/fnins.2014.00298) 25339857 PMC4186285

[11] Rilling JK Glasser MF Preuss TM Ma X Zhao T Hu X Behrens TEJ 2008 The evolution of the arcuate fasciculus revealed with comparative DTI. Nat. Neurosci. 11, 426–428. (10.1038/nn2072) 18344993

[12] Dawe RJ Bennett DA Schneider JA Vasireddi SK Arfanakis K 2009 Postmortem MRI of human brain hemispheres: T2 relaxation times during formaldehyde fixation. Magn. Reson. Med. 61, 810–818. (10.1002/mrm.21909) 19189294 PMC2713761

[13] Miller KL McNab JA Jbabdi S Douaud G 2012 Diffusion tractography of post-mortem human brains: optimization and comparison of spin echo and steady-state free precession techniques. NeuroImage 59, 2284–2297. (10.1016/j.neuroimage.2011.09.054) 22008372 PMC3314951

[14] Herzing DL 2000 Acoustics and social behavior of wild dolphins: implications for a sound society. In Hearing by whales and dolphins (eds Au WWL Fay RR Popper AN), pp. 225–272. New York, NY: Springer.

[15] Connor RC Heithaus MR Barre LM 1999 Superalliance of bottlenose dolphins. Nature 397, 571–572. (10.1038/17501)

[16] Marino L 2007 Cetaceans have complex brains for complex cognition. PLos Biol. 5, e139 (10.1371/journal.pbio.0050139) 17503965 PMC1868071

[17] Popov VV Ladygina TF Supin AY 1986 Evoked potentials of the auditory cortex of the porpoise, *Phocoena phocoena*. J. Comp. Physiol. A 158, 705–711. (10.1007/BF00603828) 3735161

[18] Sokolov VE Ladygina TF Supin AY 1972 Localization of sensory zones in the dolphin's cerebral cortex. Dokl. Akad. Nauk. SSSR 202, 490–493. 4333815

[19] Revishchin AV Garey LJ 1990 The thalamic projection to the sensory neocortex of the porpoise, *Phocoena phocoena*. J. Anat. 169, 85–102. 2384340 PMC1256959

[20] Thewissen JGM Madar SI Hussain ST 1998 Whale ankles and evolutionary relationships. Nature 395, 452 (10.1038/26656) 9774101

[21] Glezer II Jacobs MS Morgane PJ 1988 Implications of the ‘initial brain’ concept for brain evolution in Cetacea. Behav. Brain Sci. 11, 75–89. (10.1017/S0140525X0005281X)

[22] Oelschläger HA 1990 Evolutionary morphology and acoustics in the dolphin skull. In Sensory abilities of cetaceans (eds Thomas JA Kastelein RA), pp. 137–162. New York, NY: Springer.

[23] Oelschlager HHA Oelschlager JS 2009 Brain. In Encyclopedia of marine mammals, 2nd edn (eds Perrin WF Wursig B Thewissen JGM). Burlington, MA: Academic Press.

[24] Ridgway SH 1986 Physiological observations on dolphin brains. In Dolphin cognition and behavior: a comparative approach (eds Schusterman RJ Thomas JA Wood FG). Hillsdale, NJ: Lawrence Erlbaum Associates, Inc.

[25] Geraci JR Lounsbury VJ 2005 Marine mammals ashore. A field guide for strandings, 2nd edn Baltimore, MD: National Aquarium in Baltimore.

[26] Gutman DA Keifer OP Magnuson ME Choi DC Majeed W Keilholtz S Ressler KJ 2012 A DTI tractography analysis of infralimbic and prelimbic connectivity in the mouse using high-throughput MRI. NeuroImage 63, 800–811. (10.1016/j.neuroimage.2012.07.014) 22796992 PMC3488432

[27] Miller KL 2011 Diffusion imaging of whole, post-mortem human brains on a clinical MRI scanner. NeuroImage 57, 167–181. (10.1016/j.neuroimage.2011.03.070) 21473920 PMC3115068

[28] McNab JA Jbabdi S Deoni SCL Douaud G Behrens TEJ Miller KL 2009 High resolution diffusion-weighted imaging in fixed human brain using diffusion-weighted steady state free precession. NeuroImage 46, 775–785. (10.1016/j.neuroimage.2009.01.008) 19344686

[29] Buxton R 1993 The diffusion sensitivity of fast steady-state free precession imaging. Magn. Reson. Med. 19, 240–246. (10.1002/mrm.1910290212) 8429788

[30] Basser PJ Mattiello J LeBihan D 1994 Estimation of the effective self-diffusion tensor from the NMR spin echo. J. Magn. Reson. B 103, 247–254. (10.1006/jmrb.1994.1037) 8019776

[31] Behrens TEJ Johansen-Berg H Jbabdi S Rushworth MFS Woolrich MW 2007 Probabilistic diffusion tractography with multiple fibre orientations: what can we gain? NeuroImage 34, 144–155. (10.1016/j.neuroimage.2006.09.018) 17070705 PMC7116582

[32] Yeh F-C Verstynen TD Wang Y Fernandez-Miranda JC Tseng W-YI 2013 Deterministic diffusion fiber tracking improved by quantitative anisotropy. PLoS ONE 8, e80713 (10.1371/journal.pone.0080713) 24348913 PMC3858183

[33] Behrens TEJ 2003 Non-invasive mapping of connections between human thalamus and cortex using diffusion imaging. Nat. Neurosci. 6, 750–757. (10.1038/nn1075) 12808459

[34] Draganski B 2008 Evidence for segregated and integrative connectivity patterns in the human basal ganglia. J. Neurosci. 28, 7143–7152. (10.1523/JNEUROSCI.1486-08.2008) 18614684 PMC6670486

[35] Krubitzer L 2009 In search of a unifying theory of complex brain evolution. Ann. NY Acad. Sci. 1156, 44–67. (10.1111/j.1749-6632.2009.04421.x) 19338502 PMC2666944

[36] Hof PR Chanis R Marino L 2005 Cortical complexity in cetacean brains. Anat. Rec. A 287A, 1142–1152. (10.1002/ar.a.20258) 16200644

[37] Hof PR Glezer II Nimchinsky EA Erwin JM 2000 Neurochemical and cellular specializations in the mammalian neocortex reflect phylogenetic relationships: evidence from primates, cetaceans, and artiodactyls. Brain Behav. Evol. 55, 300–310. (10.1159/000006665) 10971015

[38] Geisler JH Theodor JM 2009 Hippopotamus and whale phylogeny. Nature 458, E1–E5. (10.1038/nature07776) 19295550

[39] Milinkovitch MC Berube M Palsboll PJ 1998 Cetaceans are highly derived artiodactyls. In The emergence of whales (ed. Thewissen JGM), pp. 113–131. New York, NY: Plenum Press.

[40] Butti C 2014 The cerebral cortex of the pygmy hippopotamus, *Hexaprotodon liberiensis* (Cetartiodactyla, Hippopotamidae): MRI, cytoarchitecture, and neuronal morphology. Anat. Rec. 297, 670–700. (10.1002/ar.22875) 24474726

[41] Adrian ED 1943 Afferent areas in the brain of ungulates. Brain 66, 89–103. (10.1093/brain/66.2.89)

[42] Andrews RJ Knight RT Kirby RP 1990 Evoked potential mapping of auditory and somatosensory cortices in the miniature swine. Neurosci. Lett. 114, 27–31. (10.1016/0304-3940(90)90423-7) 2116608

[43] Plogmann D Kruska D 1990 Volumetric comparison of auditory structures in the brains of European wild boars (*Sus scrofa*) and domestic pigs (*Sus scrofa* f. dom.). Brain Behav. Evol. 35, 146–155. (10.1159/000115863) 2375973

[44] Deacon TW 1990 Rethinking mammalian brain evolution. Am. Zool. 30, 629–705. (10.1093/icb/30.3.629)

[45] Oelschlager HHA 2008 The dolphin brain—a challenge for synthetic neurobiology. Brain Res. Bull. 75, 450–459. (10.1016/j.brainresbull.2007.10.051) 18331914

[46] Jerison HJ 1973 Evolution of the brain and intelligence. New York, NY: Academic Press.

[47] Thomas C Ye FQ Irfanoglu MO Modi P Saleen KS Leopold DA Pierpaoli C 2014 Anatomical accuracy of brain connections derived from diffusion MRI tractography is inherently limited. Proc. Natl Acad. Sci. USA 111, 16 574–16 579. (10.1073/pnas.1405672111) PMC4246325 25368179

[48] Kossl M Hechavarria JC Voss C Macias S Mora EC Vater M 2014 Neural maps for target range in the auditory cortex of echolating bats. Curr. Opin. Neurobiol. 24, 68–75. (10.1016/j.conb.2013.08.016) 24492081

[49] Parker J Tsagkogeorga G Cotton JA Liu Y Provero P Stupka E Rossiter SJ 2013 Genome-wide signatures of convergent evolution in echolocating mammals. Nature 502, 228–231. (10.1038/nature12511) 24005325 PMC3836225

[50] Hof PR Van der Gucht E 2007 Structure of the cerebral cortex of the humback whale, *Megaptera novaeangliae* (Cetacea, Mysticeti, Balaenaopteridae). Anat. Rec. 290, 1–31. (10.1002/ar.20407) 17441195

