# Supplementary figures and images for: Diffusion tensor imaging of dolphin brains reveals direct auditory pathway to temporal lobe
Source: Proc Biol Sci. 2015 Jul 22;282(1811):20151203. doi: 10.1098/rspb.2015.1203 (PMC4528565; doi:10.1098/rspb.2015.1203)

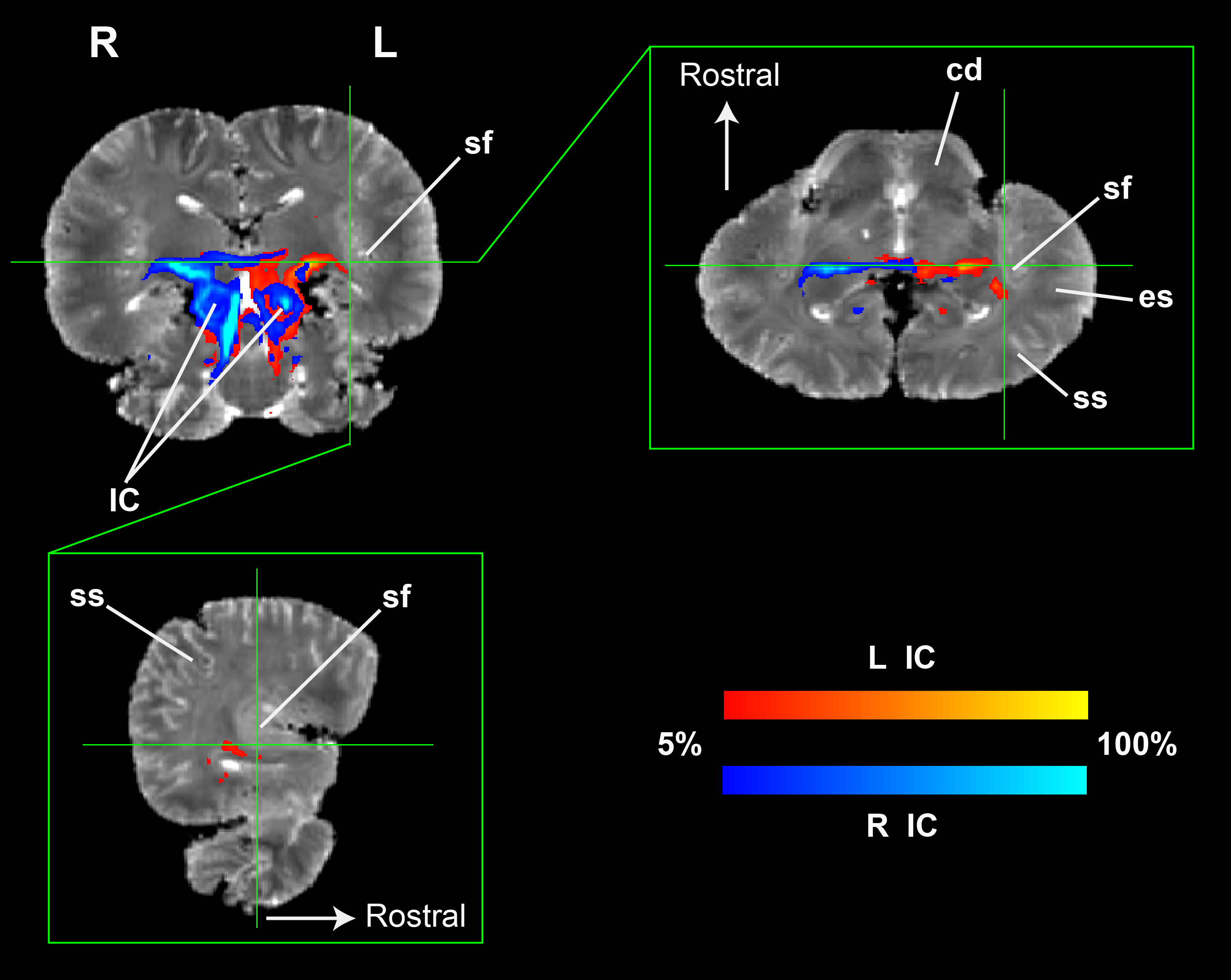

Supplement: Fig. S1. Probabilistic tractography from inferior colliculi of a pantropical dolphin (Stenella attenuata). [file rspb20151203supp1.tif]
